# Organ-Specificity of Breast Cancer Metastasis

**DOI:** 10.3390/ijms242115625

**Published:** 2023-10-26

**Authors:** Marina K. Ibragimova, Matvey M. Tsyganov, Ekaterina A. Kravtsova, Irina A. Tsydenova, Nikolai V. Litviakov

**Affiliations:** 1 Department of Experimental Oncology, Cancer Research Institute, Tomsk National Research Medical Center of the Russian Academy of Sciences, Tomsk 634009, Russia; tsyganovmm@yandex.ru (M.M.T.); zdereva.e@gmail.com (E.A.K.); tsydenova422@gmail.com (I.A.T.); nvlitv72@yandex.ru (N.V.L.); 2 Biological Institute, National Research Tomsk State University, Tomsk 634050, Russia; 3 Faculty of Medicine and Biology, Siberian State Medical University, Tomsk 634050, Russia

**Keywords:** breast cancer, organ-specific markers of metastasis, progression

## Abstract

Breast cancer (BC) remains one of the most common malignancies among women worldwide. Breast cancer shows metastatic heterogeneity with priority to different organs, which leads to differences in prognosis and response to therapy among patients. The main targets for metastasis in BC are the bone, lung, liver and brain. The molecular mechanism of BC organ-specificity is still under investigation. In recent years, the appearance of new genomic approaches has led to unprecedented changes in the understanding of breast cancer metastasis organ-specificity and has provided a new platform for the development of more effective therapeutic agents. This review summarises recent data on molecular organ-specific markers of metastasis as the basis of a possible therapeutic approach in order to improve the diagnosis and prognosis of patients with metastatically heterogeneous breast cancer.

## 1. Introduction

Breast cancer is the most frequently diagnosed malignant neoplasm in women worldwide and is expected to account for about 25% of all new cancers detected in the female population in the near future [1]. Despite the high incidence rate (2,261,419 cases in 2020 [2]), mortality rates are slowly decreasing in developed countries with the implementation of earlier diagnosis and therapy improvements [3]. Nevertheless, primary disseminated breast cancer is still diagnosed in 10% of women and the 5-year survival rate in these patients is only 25% [4].

It is the development of metastases in breast cancer, not the primary tumour, that is responsible for more than 90% of cancer deaths. According to recent studies, patients with metastatic breast cancer have bone metastases in up to 60–75% of cases, lung metastases in up to 32–37%, liver metastases in up to 32–35%, and brain metastases in up to 10% [5,6]. There is a frequency of metastases to the gastrointestinal tract in breast cancer ranging from 4% to 8% [7], and metastases to the adrenal glands are rare [8].

The detection of ovarian metastases is one of the controversial issues in the specificity of distant metastasis of breast cancer. The incidence of ovarian metastases in breast cancer patients has been shown to be 3–47%, which is mainly demonstrated during autopsies as well as prophylactic and curative oophorectomies [9,10]. Moreover, patients with breast cancer have been shown to be 3–7 times more likely to have primary ovarian cancer than ovarian metastases [11]. In addition, metastatic ovarian lesions in breast cancer sometimes mimic the clinical and histological features of primary ovarian cancer and even lose the characteristic oestrogen receptor (ER) and progesterone receptor (PR) expression levels [12,13].

In turn, the metastatic organ-specific heterogeneity of breast cancer leads to different treatment responses and patient prognosis, in particular, the 5-year overall survival (OS) in the presence of bone metastases is 22.8% [14], in the presence of lung metastasis it is 19% [15], and in the presence of liver metastasis it is 13% [16]. The median OS in the presence of ovarian metastasis is reported to be 16–38 months [17], and the 5-year survival rate is 6–26% [18]. The presence of brain metastasis in patients with breast cancer results in the shortest life expectancy [19].

**Figure 1:**
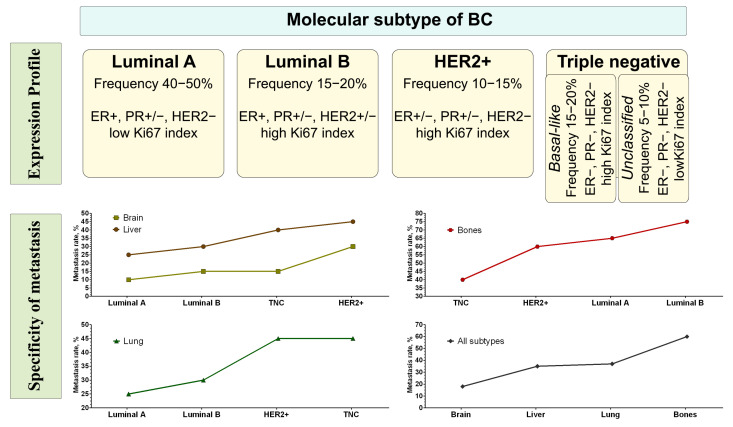
Frequency of metastasis to target organs depending on the molecular subtype of breast tumour. Note: subtypes are grouped into four categories based on the immunohistochemical expression of hormone receptors: oestrogen receptor positive (ER+), progesterone receptor positive (PR+), human epidermal growth factor receptor positive (HER2+) and triple-negative.

Different molecular subtypes of breast cancer show different metastatic organotropism. Figure 1 shows the combined literature data on the frequency of metastatic lesions to target organs depending on the molecular subtype of breast tumour [4,20].

Metastasis progression and survival prognosis may depend on specific risk factors such as the extent of lymph node involvement and tumour size. Moreover, in clinical practice, information regarding the risk of metastasis to specific target organs is tentatively determined by the molecular/histopathological subtype of the tumour (Figure 1). All these factors, however, do not allow us to fully predict the specific sites or patterns of metastasis that are characteristic of each tumour.

A hypothesis has been put forward that the primary tumour can provide insight into the organ where metastases will eventually arise. This may have a significant impact on therapeutic and screening strategies for each patient from the time of initial diagnosis. Although organotropism of breast cancer metastasis has a known “statistical” correlation [5], this process remains largely unexplained, and today there is no available diagnostic tool that can accurately predict the risk and target organ preference for each individual patient’s tumour.

The development of targeted systemic treatment has improved median overall survival in metastatic breast cancer (MBC), although many targeted treatments remain expensive and may cause harmful side effects. Promising results have been reported in small cohorts of MBC patients using combination therapy. The CLEOPATRA trial showed that for HER2+ breast cancer that overexpresses HER2 or has *ERBB2* (HER2) gene amplification, 16% of patients were progression-free at 8 years and could be effectively treated [21]. The use of a combination of CDK4 and CDK6 inhibitors (CDK4/6) with endocrine therapy for the treatment of HR+ and HER2− breast cancer improves overall survival [22], and increases the proportion of patients with a long-term response [23].

Systematic and in-depth studies of the molecular organ-specific heterogeneity of breast cancer metastasis would allow the identification of more effective agents that target metastasis suppression and contribute to improved patient outcomes.

This review brings together recent data on molecular organ-specific markers of breast cancer metastasis as the basis of a possible therapeutic approach in order to improve the diagnosis and prognosis of patients.

## 2. Metastatic Breast Cancer Signature

Regardless of the tumour type, dissemination of tumour cells precedes the initial stage of the metastasis cascade. The dissemination process includes the initial steps of the invasion and metastasis cascade, which allow malignant tumour cells to acquire properties that make it possible for them to leave the primary site and migrate to certain distant tissues [24].

One of the most important assumptions that leads scientists to study the organ-specificity of tumour metastasis, and breast cancer in particular, is the assumption that the nature of the primary tumour cell and their spread subsequently determines different metastatic properties, organotropism and response to therapy [25]. In vitro studies demonstrate that metastatic tumour cells migrate individually [26], whereby the spread of metastatic tumour cells in the body has been shown to occur as a cluster of tumour cells moving together [27].

The immediate process of tumour metastasis is a complex process that involves several sequential stages: local invasion with exit from the surrounding tissues of the primary tumour; invasion into blood or lymphatic vessels (intravasation); survival in the bloodstream as circulating tumour cells (CTCs); exit of CTCs from the circulatory system (extravasation); adaptation to the microenvironment in the form of disseminated tumour cells; transformation into cells initiating metastasis with the final formation of macrometastases [28].

Metastatic cancer includes a diverse set of cells with different genetic and phenotypic characteristics that cause differences in progression, metastasis and drug resistance [29]. Hundreds of genes determine invasive potential, with the assumption that a specific metastatic genetic signature can be identified in primary breast tumour cells [30]. Specific mutations may contribute to invasion and metastasis. Clinical genomics studies have shown that *TP53*, *CDKN2A*, *PTEN*, *PIK3CA* and *RB1* are the most predominant genes somatically altered in metastasis [31].

Examination of markers that predict metastatic progression has shown that late-stage cancers arise from different cell types, which influences the possible genetic and epigenetic alterations which contribute to metastatic progression [32]. For example, in colorectal cancer, cells expressing the L1 cell adhesion molecule (L1CAM) have chemoresistance and the ability to initiate metastasis [33].

There has been active work in this direction in the field of breast cancer. In addition to large-scale studies by The Cancer Genome Atlas (TCGA) and the International Cancer Genome Consortium (ICGC), which have characterised the molecular genetic status of primary breast cancer in detail [34,35], the sequencing of 617 breast cancer samples identified nine genes (*TP53*, *ESR1*, *GATA3*, *KMT2C*, *NCOR1*, *AKT1*, *NF1*, *RIC8A* and *RB1*) that were more frequently mutated in metastatic breast cancer compared to early breast cancer [36]. Genomic comparisons of primary tumour and metastatic tumour samples also found that metastatic clones frequently had a higher mutational load, including driver mutations and copy number aberrations, than primary tumours [37]. In some cancers, driver mutations that are identified in metastases may not be detected in the respective primary tumour [38].

Breast cancer metastases to the brain have been shown to be particularly clonally distinct, with a high number of private mutations compared to other breast cancer metastatic sites [39]. This supports the hypothesis that certain driver mutations may be specific to the organ to which cells metastasise and, in turn, may contribute to heterogeneous responses between distant metastases to different metastatic sites.

## 3. Organ-Specific Markers of Breast Cancer Metastasis to Distant Organs

To date, these features of breast cancer distant metastasis have been described in detail in the literature: clinical picture, diagnosis, biological mechanism and current approaches in the treatment of metastases to bone [4,40,41], lung [42,43], liver [44,45] and brain [46,47]. The work by Andrea R. Lim presents in detail the current relevant information on this issue [48].

However, there is scattered information on biomarkers of organ-specific metastasis in breast cancer in the literature. This section summarises the current information on biomarkers of metastasis to different target organs in breast cancer (Table 1).

## 4. Biomarker Profile of Rare Types of Breast Cancer Metastases to Distant Organs

### 4.1. Gynaecological Metastases

The work by Kutasovic J.R. (2018) [94] described in detail the clinicopathological and molecular profiling of breast cancer metastasis to gynaecological organs. The study included data from 54 female patients with breast cancer diagnosed with metastasis to gynaecological tissues between 1982 and 2015. A total of 258 metastatic foci (average of five metastases per patient (range 1–11 pcs)) were reported in these 54 patients. The most frequently involved gynaecological organs were the ovaries (46/54; 85.1%), fallopian tubes (29/54; 53.7%) and uterus (20/54; 37%). The median survival of patients was only 1.95 years.

In biomarker expression analysis, *FOXA1* and *GATA3*, key regulators of transcriptional activity, were shown to be highly expressed in primary tumours. Primary tumours also demonstrated CNA with amplification of 1q, 7q, 8q, 11q, 16p and 17q and deletion of 8p, 16q, 22q and Xq (identified in more than 50% of samples). The most frequent alterations in ovarian metastases (CNA identified in more than 50% of samples) included amplifications of 1p/q, 3p, 6p, 7p/q, 8q, 12q, 15q, 17q and 19p/q and deletions on 8p, 13p/q, 16q, 22q and Xq. The most frequent amplifications were detected at loci encoding *MDM4*, *CDK6*, *FGFR1*, *MYC*, *CCND1*, *CDK4* and *MDM2*.

In analysis of targeted sequencing data from matched primary tumours and metastases, it was shown that all cases had at least one mutation in common between the primary tumour and metastases, along with unique mutations present either only in the primary tumour (e.g., TBX3 in GM06BR) or only in the metastases (e.g., RB1, TP53 in GM74LO) [94].

### 4.2. Metastases to the Pancreas

Genetic analyses of breast cancer metastases to the pancreas are very limited due to the rarity of metastasis to this target organ. One study is presented, which is a case report that examined biomarkers of breast cancer metastasis to the pancreas.

*GATA3* expression and an *ERBB2* mutation (I767M) originating from a breast tumour were detected using immunohistochemistry and molecular diagnostics. The functional significance of this gene mutation has not been determined [95].

## 5. Genomic Profile of Breast Cancer Organ-Specific Metastasis

Understanding the nature of gene activity involved in metastasis has also been an important goal over the past few decades.

In addition to the development of high-throughput technologies in experimental and clinical oncology, many new prognostic gene markers (gene signatures or differentially expressed genes) that predict the risk of metastasis in patients with breast cancer have emerged [96]. In this section, current information on the study of the genomic profile (expression characteristics, active signalling pathways and CNA) of organ-specific metastasis in breast cancer is compiled.

In 2017, a comprehensive meta-analysis was conducted to investigate the expression of potential marker genes for metastatic breast cancer. In this paper, information on relative gene expression values was collected from 12 studies of primary breast cancer and metastatic breast cancer from the Genevestigator database (Nebion). The results of the data meta-analysis were corroborated with literature data regarding putative markers of metastatic breast cancer, and also the consistency of their reported differential expression was checked [97].

According to the results of the study, *VCAM1* seems to be the best potential marker of metastatic breast cancer, but should be validated by gene expression analysis in metastatic tissue samples where contamination by immune cells has been avoided. *FZD3* gene expression is high in metastatic tumours compared to primary tumours and this trend is supported by the literature. The high difference in *DEPDC1*, *NUSAP1*, *FOXM1* and *MUC1* gene expression observed between metastatic tissues and primary breast tumours can be considered as a prognostic marker for the development of metastases.

*COX2* gene expression is significantly reduced in metastatic tissue compared to both primary tumours and normal tissue, and can be used as a differential marker in the diagnosis of metastatic cancer. *RRM2* gene expression is decreased during the progression of metastatic breast cancer and can be proposed as a marker for monitoring progression. This study also revealed that *MMP1*, *VCAM1*, *FZD3*, *VEGFC*, *FOXM1* and *MUC1* genes can be considered as markers of breast cancer occurrence because these genes show significant differential expression in breast neoplasms compared to normal tissue [97].

In the same year, an interesting work on the comprehensive identification of molecular biomarkers in breast cancer metastases to the brain was published. In the presented study, the expression profiles of several cases were compared: 3 cases of breast cancer with brain metastasis, 16 cases of non-metastatic breast cancer and 16 cases of primary brain tumour. The genes encoding *BCL3*, *BNIP3*, *BNIP3P1*, *BRIP1*, *CASP14*, *CDC25A*, *DMBT1*, *IDH2*, *E2F1*, *MYCN*, *RAD51*, *RAD54L* and *VDR* were found to be distinctively overexpressed in mRMR with brain metastases (compared with non-metastatic breast cancer and brain tumours). Network analysis identified key pathways such as Akt, ERK1/2, NFkB and Ras at the predicted stage of activation in MBC. Genes with reduced expression in the dataset that were common to metastatic breast cancer and brain tumours included, for example, the cell line invasion markers *JUN*, *MMP3*, *TFF1* and *HAS2* [98].

In 2019, work on the identification of alternatively-activated pathways between primary and liver-metastatic breast cancer using microarray analysis data was presented. Gene expression microarray data were downloaded from the GEO database: 153 samples were in the primary breast cancer group and 43 samples were in the liver metastasis breast cancer group. Because there was a sampling imbalance between the primary cancer group and the metastatic cancer group, bootstrap analysis was performed: 43 samples were randomly selected from the primary cancer group and compared with 43 samples from the metastatic cancer group. The analysis had a total of 10 repeats.

It was shown that some signalling pathways were active in one condition (primary breast cancer or breast cancer with liver metastases) but not in both. The TnC, PHK, CAMK, NOS, ADCY, FAK2 and IP3-3K pathways were found to be active only in breast cancer metastases to the liver.

Some pathways were significantly active in both primary breast cancer and liver metastases, but the active genes were different. The CALM pathway in calcium signalling is represented by seven genes: *CALM1*, *CALM2*, *CALM3*, *CALML3*, *CALML4*, *CALML5* and *CALML6*. *CALM2* and *CALML5* were found to be significantly active in primary breast cancer, whereas *CALML3* and *CALML6* were significantly active in liver metastasis of breast cancer. The BMP pathway contained 11 different genes (*GDF5*, *GDF6*, *GDF7*, *AMH*, *BMP2*, *BMP4*, *BMP5*, *BMP6*, *BMP7*, *BMP8A* and *BMP8B*). *BMP8B* was significantly active in primary breast cancer, whereas *BMP2*, *BMP4*, *BMP5*, *BMP6* and *BMP8B* were significantly active in liver metastasis. Consequently, the pathway may be active in both primary cancer and metastatic lesions, but their mechanisms may be different. In the TGF beta-signalling pathway, the *DCN* gene was active in primary breast cancer and inhibited *TGFB* expression, but *TGFB3* was active in liver metastasis [99].

In 2020, Paul M.R. et al. presented work on the genomic landscape of metastatic breast cancer to identify preferred pathways and targets. The authors performed full-exome and shallow full-genome sequencing to identify genes and pathways preferentially mutated or altered in copy number in metastases compared to the paired primary tumours from which they arose. Seven genes were predominantly mutated in metastases: *MYLK*, *PEAK1*, *SLC2A4RG*, *EVC2*, *XIRP2*, *PALB2* and *ESR1*. The copy number of four sites was predominantly altered: deletions of *STK11* and *CDKN2A/B*, and amplifications of *PTK6* and *PAQR8*. Moreover, the presence of *PAQR8* amplification was mutually exclusive with mutations in nuclear oestrogen and progesterone receptors, suggesting a role for this marker in treatment resistance. Several pathways were preferentially mutated or altered in metastases, including mTOR, CDK/RB, cAMP/PKA, WNT, HKMT and focal adhesion. By immunohistochemical analysis, pRB was preferentially inactivated and mTORC1 and WNT signalling pathways were enhanced in metastases. These results identify several therapeutic targets that do not undergo significant mutations in primary cancer but are involved in signalling transduction in metastatic recurrence and provide a genomic basis for the efficacy of *mTORC1*, *CDK4/6* and *PARP* inhibitors in metastatic breast cancer [100].

Breast International Group (BIG) has performed genomic and transcriptomic analysis of primary breast cancer and associated metastases. The AURORA study aims to investigate the processes of the metastatic recurrence of breast cancer by performing multi-omic profiling of paired primary tumours and early metastases. Data on 381 breast cancer patients were included in the work. A driver role of somatic *GATA1* and *MEN1* mutations was found. Metastases were enriched for *ESR1*, *PTEN*, *CDH1*, *PIK3CA* and RB1 mutations, *MDM4* and *MYC* amplifications and *ARID1A* deletion. Clonality changes were observed in *ERBB2* and *RB1* driver genes [101].

In 2021, a study on the role of TFF1 in the risk of breast cancer metastasising to bone was published. A retrospective analysis of 90 surgically resected breast cancer specimens was performed. TFF1 was identified as a strictly correlated primary tumour marker of bone metastases for ER+ breast cancer. To confirm this observation, analysis of TFF1 function during ER+ breast cancer oncogenesis and metastasis to bone (MCF7 model with enhancement and inhibition of TFF1 function) was performed. It was shown that in primary tumours TFF1 expression can modulate the growth of ER+ breast cancer [102].

Additionally, a study of the gene expression profile of metastatic breast cancer depending on the target organ was published in 2021. This retrospective study included 184 metastatic tumour samples from 176 patients with breast cancer [103].

In the first step, the influence of the target organ on gene expression profiling was assessed. A total of 74 genes were identified, whose high expression was specific to the site of metastasis and independent of subtype, (*p* < 0.05): 36 bone-specific genes (*WIF1*, *IBSP*, *MMP9*, *ITGB3*, *VIT*, *HBB*, *WNT5B*, *CHAD*, *BMP2*, *EYA1*, *FOXC2*, *FZD8*, *OLFML2B*, *TGFB1*, *BMP5*, *ENPP2*, *NUDT1*, *FGF7*, *FOXC1*, *BMP8A*, *EYA4*, *RNASE2*, *SRPX*, *MME*, *LIFR*, *BAX*, *SCARA5*, *EYA2*, *XRCC3*, *LEPR*, *BCL2L1*, *NCAM1*, *SMAD3*, *RAC2*, *HOXA9*, *CKB*), 18 liver-specific genes (*ALDH1A1*, *CYP4F3*, *PCK1*, *RELN*, *AGT*, *PPARGC1A*, *HNF1A*, *CDH2*, *APOE*, *GGH*, *HGF*, *MT1G*, *CLDN1*, *UBB*, *HDAC1*, *EDNRB*, *GATA4*, *MARCO*), 12 brain-specific genes (*CRYAB*, *NRCAM*, *FGF1*, *GDF15*, *SOX2*, *GRIN1*, *RASGRF1*, *SOX10*, *CHI3L1*, *ZIC2*, *NRXN1*, *LEFTY2*) and 8 skin-specific genes (*KRT14*, *KRT5*, *S100A7*, *SERPINB5*, *MMP3*, *IL20RB*, *SFN*, *TPSAB1*). It should be noted that the authors identified three genes from the list of PAM50 genes that are associated with metastasis to bone (*FOXC1*) and skin (*KRT14* and *KRT5*).

Interestingly, the authors analysed the expression level of the identified 74 genes in 390 primary breast tumours in a publicly available dataset [104], in which three types of metastatic spread were identified: bone and visceral metasynchronous spread, bone spread only, and visceral metastasis only. Visceral metastases included distant metastases to the lungs, liver and brain.

Among the 74 genes, 26 genes (35.1%) were found to be significantly associated with the type of metastatic spread, including 5 bone-specific genes (*CHAD*, *EYA1*, *TGFB1*, *BAX* and *HOXA9*) whose high expression was associated only with bone metastasis, 4 bone-specific genes (*WIF1*, *VIT*, *FOXC2* and *MME*) whose high expression was associated with bone and visceral metastasis, 2 brain-specific genes (*FGF1* and *SOX2*) whose high expression was associated with bone and visceral metastasis, 2 brain-specific genes (*RASGRF1* and *CHI3L1*) whose high expression was associated with metastasis to internal organs only, and 2 liver-specific genes (*GGH* and *MARCO*) whose high expression was associated with visceral metastasis only. This result suggests that certain genes may also indicate an organ-specific type of metastatic spread in the analysis of primary tumours [103].

Full title, acronyms and location of all genes (identified and described) in the review are presented in Table S1.

## 6. Conclusions

Metastatic progression represents a major therapeutic challenge, whereby unpredictable tumour heterogeneity both between patients and within each tumour becomes a major obstacle in the search for a rational therapeutic approach.

The accumulated knowledge on the genomics of breast cancer over the last decade has significantly increased the understanding of intratumoural heterogeneity, which is now considered to be a driving force for cancer progression. In this context, the knowledge and understanding of metastatic breast cancer is somewhat behind that of primary cancer [105].

**Figure 2:**
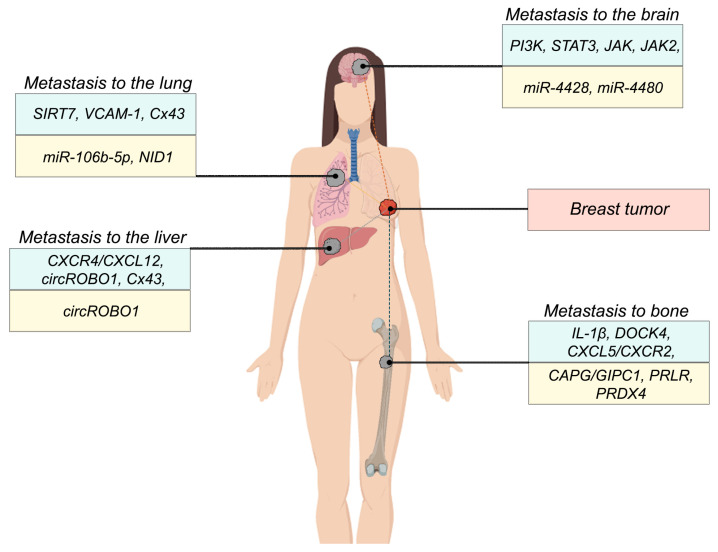
Biomarkers of organ-specificity of distant metastasis occurrence in breast cancer. Note: experimentally confirmed metastasis markers are highlighted in light blue, primary tumour metastasis markers are highlighted in light yellow.

According to the available literature, the present review summarises information on biomarkers of metastasis to different target organs in breast cancer. Figure 2 shows schematically the markers of organ-specificity of metastases development in breast cancer.

In turn, understanding the mechanism of heterogeneity, including in the context of the organospecificity of metastatic potential in breast cancer, is crucial for the development of new effective diagnostic and prognostic strategies. However, additional studies are needed to further validate the identified genes and molecular mechanisms for future clinical applications.

## Figures and Tables

**Table 1:** Markers of organ-specific metastasis in breast cancer.

Marker	Description	Source
**BONES**		
*P1NP*, *CTX*, *1-CTP*	Patients with high serum levels of *P1NP*, *CTX* and *1-CTP* have been shown to have a high risk of metastasising to bone soon after diagnosis (*p* = 0.006, *p* = 0.009, *p* = 0.008, respectively).	[49]
*IL-1β*	In preclinical experimental mouse models, *IL-1β* inhibitors have been shown to prevent the development of bone metastases.	[50]
*CAPG/GIPC1*	The identification of *CAPG* and *GIPC1* in primary tumour samples (by IHC) was a strong prognostic indicator for the development of bone metastases of breast cancer. Cox regression analysis showed that control patients were more likely to develop first distant recurrence in bone (hazard ratio [HR] = 4.5, 95% confidence interval [CI] = 2.1 to 9.8, *p* < 0.001) and die (HR for overall survival = 1.8, 95% CI = 1.01 to 3.24, *p* = 0.045) if both proteins were highly expressed in the primary tumour.	[51]
*PRLR*	High *PRLR* expression in primary breast tumour is associated with shorter time to metastasis (*p* = 0.03).	[52]
*PRDX4*	High expression of *PRDX4* in primary breast tumour is associated with metastasis within 5 years.	[53]
*PAK4*	*PAK4* enhances the invasive potential of ERα-positive breast cancer cells in vitro and promotes metastasis in vivo. The status of the nuclear *PAK4* (*nPAK4*) scores was significantly higher in the bone metastatic breast cancer group than in the non-bone metastatic breast cancer group (*p* = 2.22 × 10^−9^).	[54]
*MAF*	*MAF* is a molecular target for the prevention or treatment of bone metastases because *MAF* accumulation (16q23 amplification) plays a role in bone colonisation. 16q23 gain copy number alterations (CNA) encoding the transcription factor *MAF* mediate breast cancer bone metastasis through PTHrP control. 16q23 gain (hazard ratio (HR) for bone metastasis = 14.5, 95% confidence interval (CI) = 6.4 to 32.9, *p* < 0.001) as well as *MAF* overexpression (HR for bone metastasis = 2.5, 95% CI = 1.7 to 3.8, *p* < 0.001) in primary breast tumours were specifically associated with risk of metastasis to bone but not to other organs.	[55,56]
*DOCK4*	In a triple-negative MDA-MB-231 cell line model, *DOCK4* was identified as a biomarker of bone metastasis in early stages of breast cancer. Adjusted Cox regression analyses showed that high *DOCK4* expression in the control arm was significantly prognostic for first recurrence in bone (HR 2.13, 95%CI 1.06–4.30, *p* = 0.034) (a clinical validation). High *DOCK4* expression was not associated with metastasis to non-skeletal sites when these were assessed collectively.	[57]
*CENPF*	*CENPF* promotes breast cancer metastasis to bone by activating PI3K-AKT-mTORC1 signalling and represents a novel therapeutic target for breast cancer treatment.	[58]
*MMP9*, *MMP13*, *TNFAIP6*, *CD200*, *DHRS3*, *ASS1*, *VIM*	Together, they can be considered as specific prognostic markers of metastasis to bone in primary breast cancer. The relative expression of *MMP9*, *MMP13*, *TNFAIP6* and *CD200* were significantly up-regulated (*p* < 0.05), while *DHRS3*, *ASS1* and *VIM* were significantly down-regulated in the bone metastasis compared with lung and liver metastasis (*p* < 0.05).	[59]
*miR-200*, *-128*, *-99a*, *-29b*, *-600*, *-34*, *-30*, *let-7 miRNA*	These miRs act as tumour suppressors and inhibit breast cancer metastasis to bone.	[60,61]
*miR-21*	Exosomal *miR-21* derived from SCP28 cells promotes osteoclastogenesis through regulation of *PDCD4* protein levels. The level of *miR-21* is significantly higher in serum exosomes of breast cancer patients with bone metastases than in other subpopulations.	[62]
*CXCL5/CXCR2*	*CXCL5* stimulates proliferation of breast cancer cells and their colonisation in bone. Inhibition of its *CXCR2* receptor with an antagonist blocks the proliferation of metastatic cells. *CXCL5* and CXCR2 inhibitors may be effective in the treatment of tumours with metastasis to bone.	[63]
*RANKL/RANK*	*RANKL/RANK* regulates breast cancer cell migration. *RANKL* acts as a chemoattractive agent on tumour cells which overexpress one of its receptors. Blocking signalling by AMG161 (IgG1) reduces micrometastasis formation in bone marrow in vivo. Daily subcutaneous injections of 1.5 mg/kg AMG161 antibody to MDA-MB231RANK tumour-bearing animals reduced bone micrometastases and early bone marrow colonization without affecting lung micrometastasis.	[64]
*CXCL-12*	HIF signalling transduction in osteoporosis precursor cells increases blood levels of *CXCL-12*, promoting metastasis to bone.	[65]
*ESR1*	Mutations in the *ESR1* gene have been observed in bone metastases, suggesting a potential causative role. In this study, bone metastases from breast cancer (n = 231) were analysed for *ESR1* mutation. Activating *ESR1* mutations were identified in 27 patients (12%). The most frequent mutation was p.D538G (53%), no mutations were found in exon 4 (K303) or 7 (S463). Metastatic breast cancer with activating mutations of *ESR1* had a higher Ki67 labelling index than primary luminal cancers (median 30%, ranging from 5 to 60% with 85% of cases revealing ≥ 20% Ki67-positive cells).	[66]
*ANGPTL2*	*ANGPTL2* increases breast cancer cell metastasis to bone by enhancing *CXCR4* signal transduction.	[67]
**LUNGS**		
*miR-106b-5p*	It is an independent predictor of lung metastases (based on the expression level in the primary tumour). *MiR-106b-5p* promotes lung metastasis by suppressing *CNN1* and activating the Rho/ROCK1 pathway.	[68,69]
*SIRT7*	*SIRT7* counteracts TGFβ signalling and inhibits breast cancer metastases to the lung.	[70]
Tumour stem cells (TSCs) (CD44hi CD36+)	The formation of lung metastases is associated with TSC function, metabolic changes and immune response. Lung metastasis can be mediated by TSCs with CD44hi CD36+ phenotype.	[71]
*NID1*	Secretome analysis of lung metastases of breast cancer has shown that Nidogen 1 (*NID1*) is associated with poor treatment outcomes. *NID1* promotes lung metastasis of breast cancer by increasing the motility of tumour cells and promoting their adhesion to the endothelium, thereby compromising its integrity and promoting angiogenesis.	[72]
*EGFR*	EGFR inhibition successfully blocks circulating tumour cells (by immunohistochemistry) clustering and triple-negative breast cancer metastasis to the lung.	[73]
*VCAM-1*	*VCAM-1* can be considered as a potential therapeutic target in lung metastasis of breast cancer. Selective inhibition of *VCAM-1* has been successfully used to suppress the development of metastases. The experimental results showed that the SCB-loaded nanoparticles (SN) could greatly improve the oral delivery and suppress breast cancer metastasis to the lung. The cell migration and invasion abilities of metastatic 4T1 breast cancer cells were obviously inhibited by SN. Moreover, the VCAM-1 expression on 4T1 cells was significantly reduced by SN, and the binding ratio of RAW 264.7 cells to 4T1 cells was significantly decreased from 47.4% to 3.2%. Furthermore, the oral bioavailability of SCB was greatly increased 13-fold under the effect of SN, and the biodistribution in major organs was markedly improved.	[74]
*DKK1*	In patients with breast cancer, low serological levels of *DKK1* are associated with the risk of developing lung metastases.	[75]
*Connexin43* (*Cx43*)	Mice injected with Cx43-shCx43-inhibited tumour cells exhibited more lung metastases compared to parental MDA-MB-231 cells. This observation was confirmed by qPCR analysis of human 18S RNA levels in secondary metastatic sites in the lungs. Higher levels of human 18S RNA were found in the lungs of mice injected with shCx43 cells compared to the lungs of mice injected with parental MDA-MB-231 cells. This observation indicates that suppression of *Cx43* increases the metastatic potential of MDA-MB-231 cells.	[76]
**LIVER**		
*Connexin43* (*Cx43*)	Metastatic foci in the liver were almost absent in mice inoculated with parental MDA-MB-231 cells or Cx43D cells by week 9, compared to those clearly observed in mice inoculated with shCx43 cells. This result is consistent with the increased levels of human 18S RNA in the livers of mice inoculated with shCx43 cells. Inhibition of *Cx43* induced metastasis of MDA-MB-231 cells to lung and liver at week 9, when the original MDA-MB-231 cells had not yet metastasised. These findings correlate with increased tumour volume and decreased survival of xenograft mice in vivo.	[76]
*CXCR4/CXCL12*	*CXCR4* inhibition doubles the response to immune checkpoint blockers in mice with metastatic triple-negative breast cancer (TNBC). *CXCL12/CXCR4*-mediated desmoplasia in metastatic breast cancer promotes immunosuppression and is a potential target to overcome therapeutic resistance to immune checkpoint blockade in MBC patients.	[77]
*PDK1*	*PDK1*-dependent metabolic reprogramming is a key regulation of metabolism and metastasis to the liver in breast cancer. *PDK1* is particularly required for metabolic adaptation to nutrient restriction and hypoxia as a HIF1α target of metastatic cells in the liver.	[78]
*circRNA hsa_circ_0008324* (*circEZH2*)	*CircEZH2* enhances oncogenesis and metastasis in vitro and in vivo by activating KLF5 protein expression, which in turn activates CXCR4 transcription, leading to the initiation of the EMT programme in breast cancer.	[79]
*circRNA hsa_circ_0124696* (*circROBO1*)	Increased expression of *circROBO1* was found in liver metastases in breast cancer and correlated with poor prognosis. Knockdown of *circROBO1* strongly inhibited proliferation, migration and invasion of RRM cells, whereas *circROBO1* overexpression showed opposite effects. *circROBO1* overexpression promoted tumour growth and metastasis to the liver in vivo.	[80]
*Lyn* (*Src-family kinase*)	The Lyn-selective kinase inhibitor, bafetinib (INNO-406), reduces claudin-2 expression and suppresses breast cancer metastasis to the liver.	[81]
*PPFIA1*	*PPFIA1* is activated in breast cancer metastasis to the liver and is a potentially unfavourable prognostic sign of metastases development. Kaplan–Meier plotter results showed that although high *PPFIA1* expression was generally associated with reduced distant metastasis-free survival in oestrogen receptor+ patients, subgroup analysis only confirmed significant association in an oestrogen receptor+/N− (node-negative) group (median survival, high *PPFIA1* group vs. low *PPFIA1* cohort: 191.21 vs. 236.22 months, hazard ratio: 2.23, 95% confidence interval: 1.42–3.5, *p* < 0.001), but not in an oestrogen receptor+/N+ (nodal positive) group (hazard ratio: 1.63, 95% confidence interval: 0.88–3.03, *p* = 0.12). In oestrogen receptor patients, there was no association between *PPFIA1* expression and distant metastasis-free survival, regardless of Nm (mixed nodal status), N− or N+ subgroups. In bc-GenExMiner 4.0 programme using the Nottingham Prognostic Index and Adjuvant! Online-adjusted analysis validated the independent prognostic value of *PPFIA1* in relation to the risk of metastasis in patients with oestrogen receptor+/N−.	[82]
*ESR1*, *AKT1*, *ERBB2*, *FGFR4*	*ESR1* (20%), *AKT1* (8%), *ERBB2* (7%) and *FGFR4* (4%) were identified as driver genes for breast cancer metastasis.	[83]
**BRAIN**		
*PI3K*	Activation of *PI3K* was found in a large proportion (77%) of brain metastases in patients with breast cancer, and activation of PI3K-Akt signalling in such metastases was associated with poor outcomes. Pharmacological inhibition of *PI3K* activity was found to attenuate the expression of *PD-L1*, *CTLA4* and *CSF1* genes, as well as the infiltration of metastatic breast cancer cells into the brains of mice.	[84,85]
*CDK4 u CDK6*	Abemaciclib, an inhibitor of the cyclin-dependent kinases *CDK4* and *CDK6*, has shown potential for the treatment of brain metastases in patients with breast cancer. The combination of abemaciclib with endocrine therapy was effective in patients with HER2-negative breast cancer and brain metastases, and 38% of patients had a reduction in metastatic tumour burden.	[86]
*STAT3*	The *STAT3* inhibitor silibinin, which penetrates the blood–brain barrier, impairs the viability of brain metastases in both mice and humans. This inhibitor is thought to block the growth of brain metastases by targeting *STAT3* in tumour-associated astrocytes, thereby weakening their interaction with tumour cells and microglia.	[87]
*JAK*, *JAK2*	The *JAK* inhibitor ruxolitinib limits the growth of primary brain tumours and also reduces the number of tumour-associated astrocytes in mice. JAK2/STAT3 signal transduction is hyperactivated when breast cancer metastasises to the brain. Inhibition of *JAK2* results in reduced brain metastasis in vivo, suggesting that *JAK2* may be a promising therapeutic target.	[88,89]
*COX2*	*COX2* can promote *MMP1* expression, which is significantly correlated with brain metastasis. In addition, *COX2* and prostaglandin activate astrocytes to release chemokine ligand, promoting self-renewal of tumour stem cells or tumour-initiating cells in the brain.	[90]
*FABP7*	*FABP7* is a key regulator of metabolism in HER2+ breast cancer metastasis to the brain. *FABP7* has been shown to be required for the activation of key metastatic genes and pathways, such as integrins-Src and VEGFA, as well as for the growth of HER2+ breast cancer cells in the brain microenvironment in vivo.	[91]
*miR-4428*, *miR-4480*	In a study of microRNAs in patients with advanced breast cancer with brain metastases, it was shown that the determination of *miR-4428* and *miR-4480* in serum may be useful as prognostic biomarkers. A total of 51 serum samples from patients with breast cancer and brain metastasis, and 28 serum samples from controls without brain metastasis were obtained. Two miRNAs, miR-4428 and miR-4480 could significantly distinguish patients with brain metastasis, with area under the receiver operating characteristic curve (AUC) values of 0.779 and 0.781, respectively, while a combination of miR-4428 and progesterone receptor had an AUC value of 0.884.	[92]
*PLVAP*	*PLVAP* staining was observed not only in isolated brain microvessels but also in brain metastases in breast cancer. Immune labelling for *PLVAP* was performed in 4T1 TNBC culture, where clear expression of this protein was observed.	[93]

## Data Availability

Not applicable.

## Supplementary Materials

The following supporting information can be downloaded at: https://www.mdpi.com/article/10.3390/ijms242115625/s1.
