# Supplementary material for: Organ-Specificity of Breast Cancer Metastasis
Source: Int J Mol Sci. 2023 Oct 26;24(21):15625. doi: 10.3390/ijms242115625 (PMC10650169; doi:10.3390/ijms242115625)
Supplement: Supplementary file 1 [file ijms-24-15625-s001.zip › ijms-2663076-SI.pdf]

Table S1. Full title, acronyms and location of all genes (identified and described) presented in the review.

Gene	Full title	Location
AGT	Angiotensinogen	1q42.2
AKT1	V-Akt Murine Thymoma Viral Oncogene Homolog 1	14q32.33
AMH	Anti-Mullerian Hormone	19p13.3
ANGPTL2	Angiopoietin-Related Protein 2	9q33.3
APOE	Apolipoprotein E	19q13.32
ASS1	Argininosuccinate Synthase 1	9q34.11
BAX	BCL2 Associated X, Apoptosis Regulator	19q13.33
BCL2L1	BCL2 Like 1	20q11.21
BCL3	BCL3 Transcription Coactivator	19q13.32
BMP2	Bone Morphogenetic Protein 2	20p12.3
BMP4	Bone Morphogenetic Protein 4	14q22.2
BMP5	Bone Morphogenetic Protein 5	6p12.1
BMP6	Bone Morphogenetic Protein 6	6p24.3
BMP7	Bone Morphogenetic Protein 7	20q13.31
BMP8A	Bone Morphogenetic Protein 8a	1p34.3
BMP8B	Bone Morphogenetic Protein 8b	1p34.2
BNIP3	BCL2 Interacting Protein 3	10q26.3
BNIP3P1	BCL2 Interacting Protein 3 Pseudogene 1	14q12
BRIP1	BRCA1 Interacting Helicase 1	17q23.2
CALM1	Calmodulin 1	14q32.11
CALM2	Calmodulin 2	2p21
CALM3	Calmodulin 3	19q13.32
CALML3	Calmodulin Like 3	10p15.1
CALML4	Calmodulin Like 4	15q23
CALML5	Calmodulin Like 5	10p15.1
CALML6	Calmodulin Like 6	1p36.33
CAPG	Capping Actin Protein, Gelsolin Like	2p11.2
CASP14	Caspase 14	19p13.12
CCND1	Cyclin D1	11q13.3
CD200	CD200 Molecule	3q13.2
CDC25A	Cell Division Cycle 25A	3p21.31
CDH2	Cadherin 2	18q12.1
CDK4	Cyclin Dependent Kinase 4	12q14.1
CDK6	Cyclin Dependent Kinase 6	7q21.2
CDKN2A	Cyclin Dependent Kinase Inhibitor 2A	9p21.3
CENPF	Centromere Protein F	1q41
CHAD	Chondroadherin	17q21.33
CHI3L1	Chitinase 3 Like 1	1q32.1
CKB	Creatine Kinase B	14q32.33
CLDN1	Claudin 1	3q28

COX2	Long intergenic noncoding RNA COX2	1q25.2
CRYAB	Crystallin Alpha B	11q23.1
CTX	Cytochrome P450 Family 27 Subfamily A Member 1	2q35
CXCL12	C-X-C Motif Chemokine Ligand 12	10q11.21
CXCL5	C-X-C Motif Chemokine Ligand 5	4q13.3
CXCR2	C-X-C Motif Chemokine Receptor 2	2q35
CXCR4	C-X-C Motif Chemokine Receptor 4	2q22.1
CYP4F3	Cytochrome P450 Family 4 Subfamily F Member 3	19p13.12
DEPDC1	DEP Domain Containing 1	1p31.3
DHRS3	Dehydrogenase/Reductase 3	1p36.21
DKK1	Dickkopf WNT Signaling Pathway Inhibitor 1	10q21.1
DMBT1	Deleted In Malignant Brain Tumors 1	10q26.13
DOCK4	Dedicator Of Cytokinesis 4	7q31.1
E2F1	E2F Transcription Factor 1	20q11.22
EDNRB	Endothelin Receptor Type B	13q22.3
EGFR	Epidermal Growth Factor Receptor	7p11.2
ENPP2	Ectonucleotide Pyrophosphatase/Phosphodiesterase 2	8q24.12
ERBB2	Erb-B2 Receptor Tyrosine Kinase 2	17q12
ESR1	Estrogen Receptor 1	6q25.1
EVC2	EvC Ciliary Complex Subunit 2	4p16.2
EYA1	EYA Transcriptional Coactivator And Phosphatase 1	8q13.3
EYA2	EYA Transcriptional Coactivator And Phosphatase 2	20q13.12
EYA4	EYA Transcriptional Coactivator And Phosphatase 4	6q23.2
FABP7	Fatty Acid Binding Protein 7	6q22.31
FGF1	Fibroblast Growth Factor 1	5q31.3
FGF7	Fibroblast Growth Factor 7	15q21.2
FGFR1	Fibroblast Growth Factor Receptor 1	8p11.23
FGFR4	Fibroblast Growth Factor Receptor 4	5q35.2
FOXA1	Forkhead Box A1	14q21.1
FOXC1	Forkhead Box C1	6p25.3
FOXC2	Forkhead Box C2	16q24.1
FOXM1	Forkhead Box M1	12p13.33
FZD3	Frizzled Class Receptor 3	8p21.1
FZD8	Frizzled Class Receptor 8	10p11.21
GATA3	GATA Binding Protein 3	10p14
GATA4	GATA-binding Protein 4	8p23.1
GDF15	Growth Differentiation Factor 15	19p13.11
GDF5	Growth Differentiation Factor 5	20q11.22
GDF6	Growth Differentiation Factor 6	8q22.1
GDF7	Growth Differentiation Factor 7	2p24.1
GGH	Gamma-Glutamyl Hydrolase	8q12.3
GRIN1	Glutamate Ionotropic Receptor NMDA Type Subunit 1	9q34.3
HAS2	Hyaluronan Synthase 2	8q24.13
HBB	Hemoglobin Subunit Beta	11p15.4

HDAC1	Histone Deacetylase 1	1p35.2-p35.1
HGF	Hepatocyte Growth Factor	7q21.11
HNF1A	HNF1 Homeobox A	12q24.31
HOXA9	Homeobox A9	7p15.2
IBSP	Integrin-binding Sialoprotein	4q22.1
IDH2	Isocitrate Dehydrogenase (NADP(+)) 2	15q26.1
IL-1β	Interleukin 1 Beta	2q14.1
IL20RB	Interleukin 20 Receptor Subunit Beta	3q22.3
ITGB3	Integrin Subunit Beta 3	17q21.32
JAK1	Janus Kinase 1	1p31.3
JAK2	Janus Kinase 2	9p24.1
JUN	Jun Proto-Oncogene, AP-1 Transcription Factor Subunit	1p32.1
KMT2C	Lysine Methyltransferase 2C	7q36.1
KRT14	Keratin 14	17q21.2
KRT5	Keratin 5	12q13.1
LEFTY2	Left–Right Determination Factor 2	1q42.12
LEPR	Leptin Receptor	1p31.3
LIFR	LIF Receptor Subunit Alpha	5p13.1
MAF	MAF BZIP Transcription Factor	16q23.2
MARCO	Macrophage Receptor With Collagenous Structure	2q14.2
MDM2	MDM2 Proto-Oncogene	12q15
MDM4	MDM4 Regulator Of P53	1q32.1
MME	Membrane Metalloendopeptidase	3q25.2
MMP1	Matrix Metallopeptidase 1	11q22.2
MMP13	Matrix Metallopeptidase 13	11q22.2
MMP3	Matrix Metallopeptidase 3	11q22.2
MMP9	Matrix Metallopeptidase 9	20q13.12
MT1G	Metallothionein 1G	16q13
MUC1	Mucin 1, Cell Surface Associated	1q22
MYC	MYC Proto-Oncogene, BHLH Transcription Factor	8q24.21
MYCN	MYCN Proto-Oncogene, BHLH Transcription Factor	2p24.3
MYLK	Myosin Light Chain Kinase	3q21.1
NCAM1	Neural Cell Adhesion Molecule 1	11q23.2
NCOR1	Nuclear Receptor Corepressor 1	17p12-p11.2
NF1	Neurofibromin 1	17q11.2
NID1	Nidogen 1	1q42.3
NRCAM	Neuronal Cell Adhesion Molecule	7q31.1
NRXN1	Neurexin 1	2p16.3
NUDT1	Nudix Hydrolase 1	7p22.3
NUSAP1	Nucleolar And Spindle Associated Protein 1	15q14
OLFML2B	Olfactomedin-like 2B	1q23.3
PAK4	P21 (RAC1) Activated Kinase 4	19q13.2
PALB2	Partner And Localizer Of BRCA2	16p12.2

PCK1	Phosphoenolpyruvate Carboxykinase 1	20q13.31
PDK1	Pyruvate Dehydrogenase Kinase 1	2q31.1
PEAK1	Pseudopodium Enriched Atypical Kinase 1	15q24.3
PIK3CA	Phosphatidylinositol-4,5-Bisphosphate 3-Kinase Catalytic Subunit Alpha	3q26.32
PLVAP	Plasmalemma Vesicle Associated Protein	19p13.11
PPARGC1A	PPARG Coactivator 1 Alpha	4p15.2
PPFIA1	PTPRF Interacting Protein Alpha 1	11q13.3
PRDX4	Peroxiredoxin 4	Xp22.11
PRLR	Prolactin Receptor	5p13.2
PTEN	Phosphatase And Tensin Homolog	10q23.31
RAC2	Rac Family Small GTPase 2	22q13.1
RAD51	RAD51 Recombinase	15q15.1
RAD54L	RAD54 Like	1p34.1
RANK	TNF Receptor Superfamily Member 11a	18q21.33
RANKL	TNF Superfamily Member 11	13q14
RASGRF1	Ras Protein Specific Guanine Nucleotide Releasing Factor 1	15q25.1
RB1	RB Transcriptional Corepressor 1	13q14.2
RELN	Reelin	7q22.1
RIC8A	RIC8 Guanine Nucleotide Exchange Factor A	11p15.5
RNASE2	Ribonuclease A Family Member 2	14q11.2
RRM2	Ribonucleotide Reductase Regulatory Subunit M2	2p25.1
S100A7	S100 Calcium Binding Protein A7	1q21.3
SCARA5	Scavenger Receptor Class A Member 5	8p21.1
SERPINB5	Serpin Family B Member 5	18q21.33
SFN	Stratifin	1p36.11
SIRT7	Sirtuin 7	17q25.3
SLC2A4RG	SLC2A4 Regulator	20q13.33
SMAD3	SMAD Family Member 3	15q22.33
SOX10	SRY-Box Transcription Factor 10	22q13.1
SOX2	SRY-Box Transcription Factor 2	3q26.33
SRPX	Sushi-repeat Containing Protein Xlinked	Xp11.4
STAT3	Signal Transducer And Activator Of Transcription 3	17q21.2
STK11	Serine/Threonine Kinase 11	19p13.3
TFF1	Trefoil Factor 1	21q22.3
TGFB1	Transforming Growth Factor Beta 1	19q13.2
TNFAIP6	TNF Alpha Induced Protein 6	2q23.3
TP53	Tumor Protein P53	17p13.1
TPSAB1	Tryptase Alpha/Beta 1	16p13.3
UBB	Ubiquitin B	17p11.2
VCAM1	Vascular Cell Adhesion Molecule 1	1p21.2
VDR	Vitamin D Receptor	12q13.11
VEGFC	Vascular Endothelial Growth Factor C	4q34.3
VIM	Vimentin	10p13

VIT	Vitrin	2p22.2
WIF1	WNT Inhibitory Factor 1	12q14.3
WNT5B	Wnt Family Member 5B	12p13.33
XIRP2	Xin Actin Binding Repeat Containing 2	2q24.3
XRCC3	X-ray Repair Cross-complementing 3	14q32.33
ZIC2	Zic Family Member 2	13q32.3
